# Supplementary material for: Magnetoelectric effect and phase transitions in CuO in external magnetic fields
Source: Nat Commun. 2016 Jan 18;7:10295. doi: 10.1038/ncomms10295 (PMC4735624; doi:10.1038/ncomms10295)
Supplement: Supplementary Information — Supplementary Figures 1-9, Supplementary Notes 1-4 and Supplementary References [file ncomms10295-s1.pdf]

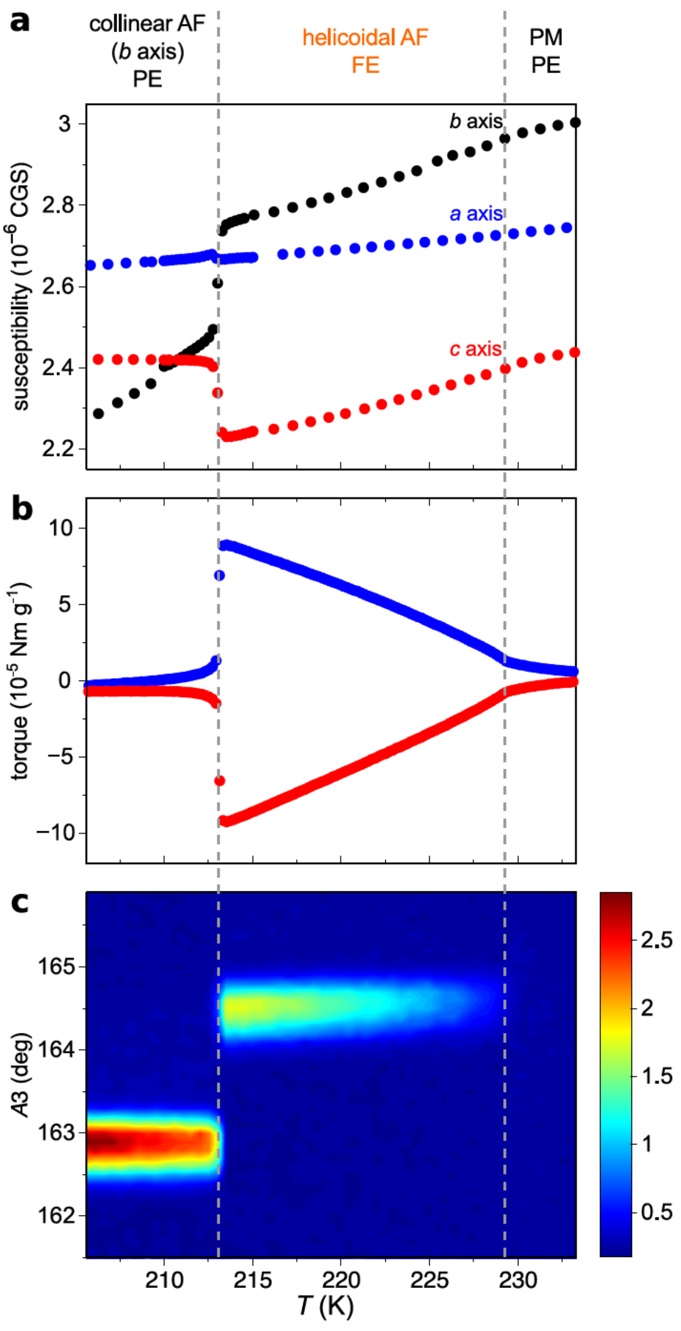


**Supplementary Figure 1│ Temperature-induced transitions in CuO.** (**a**) Temperature dependence of the low-field (0.1 T) magnetic susceptibility along the principle crystallographic directions. (**b**) Temperature dependence of the magnetic torque in two distinct orthogonal magnetic directions within the *ac* plane, corresponding to the *easy* and the *hard* magnetic axes in the paramagnetic state (those directions, in blue and in red colours, are close to but do not coincide with the crystallographic *a* and *c* axes, correspondingly). (**c**) Commensurate to incommensurate temperature-induced phase transition: neutron rocking curves (rotation of the crystal around the diffractometer vertical axis) as a function of temperature. The position A3 = 162.8° corresponds to $q$ = (0.500 0.000 –0.500), whereas A3 = 164.5° is $q$ = (0.509 0.000 –0.483). The scattered intensity is color coded between 0 and 2.86x$10^3$ counts per 6s.

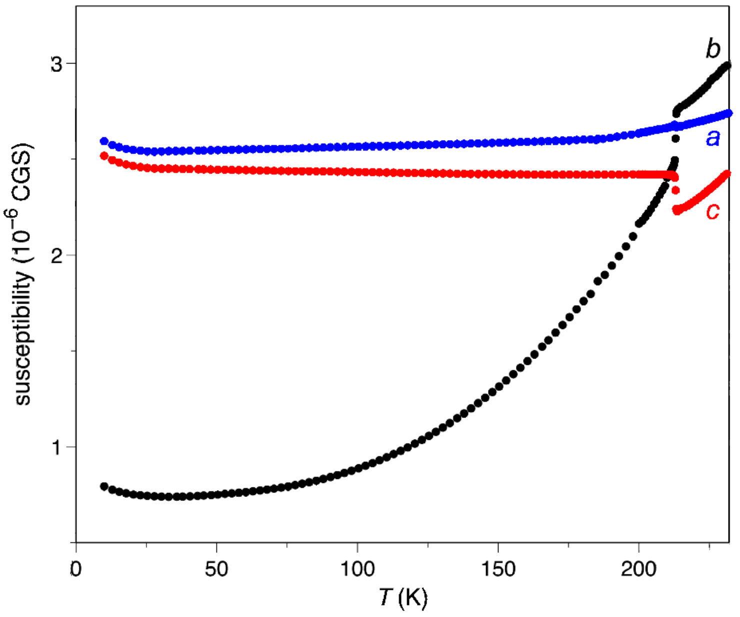


**Supplementary Figure 2│ Low-temperature low-field magnetic susceptibility.** Temperature dependence of the low field (0.1 T) magnetic susceptibility along the principle crystallographic directions.

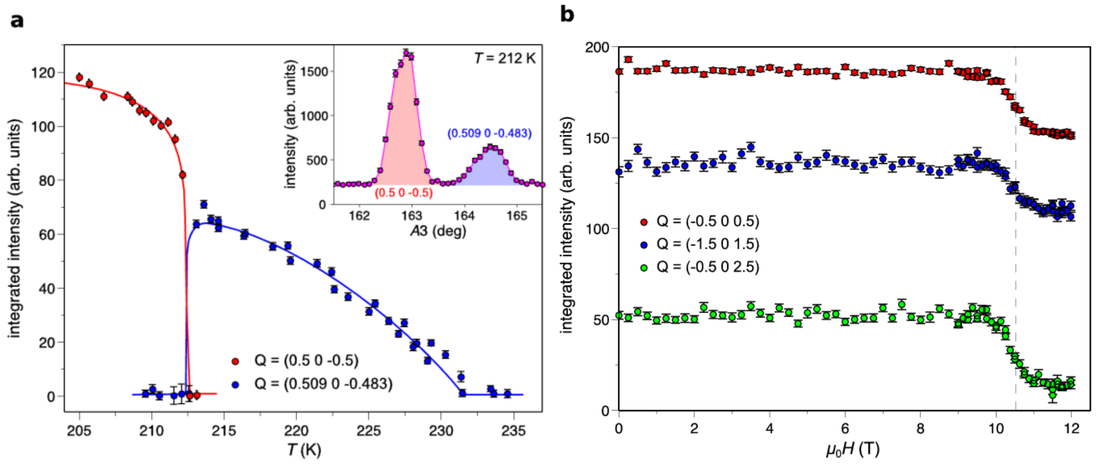


**Supplementary Figure 3│ Temperature and field variation of some selected neutron reflections** (**a**) Temperature evolution of both an incommensurate magnetic peak $q$ = (0.509 0.000 –0.483) and a commensurate one $q$ = (0.500 0.000 –0.500) at zero magnetic field. The inset shows A3 scan at 212 K. (**b**) Field-induced transition at 10 K as seen from the change in the integrated intensities suggesting a reorientation of the magnetic moments.

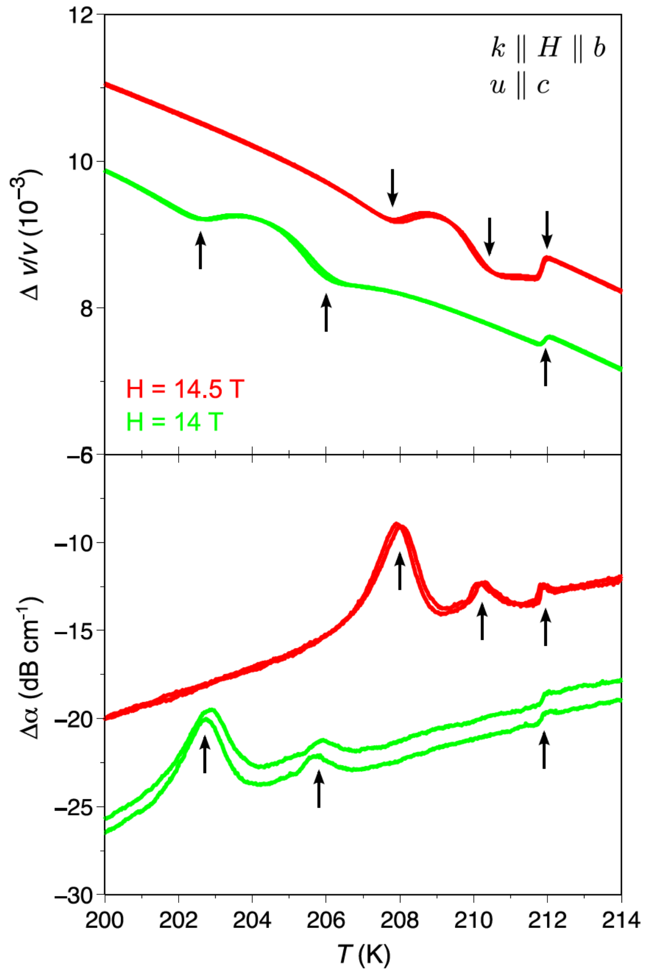


**Supplementary Figure 4│ Sound velocity and sound attenuation.** Temperature dependence of the sound velocity $\Delta v/v$ and the sound attenuation $\Delta\alpha$, of the transverse ultrasonic wave with wave vector **k** propagating along the *b* axis with polarization **u** directed along the *c* axis (**k**||**H**||*b* and **u**||*c*) measured at different applied magnetic fields. The ultrasound frequency was 110 MHz. The data at different *H* are shifted arbitrary along the *y*-axis for clarity. Results for up and down temperature sweeps are shown. The arrows indicate the temperatures at which anomalies are observed.

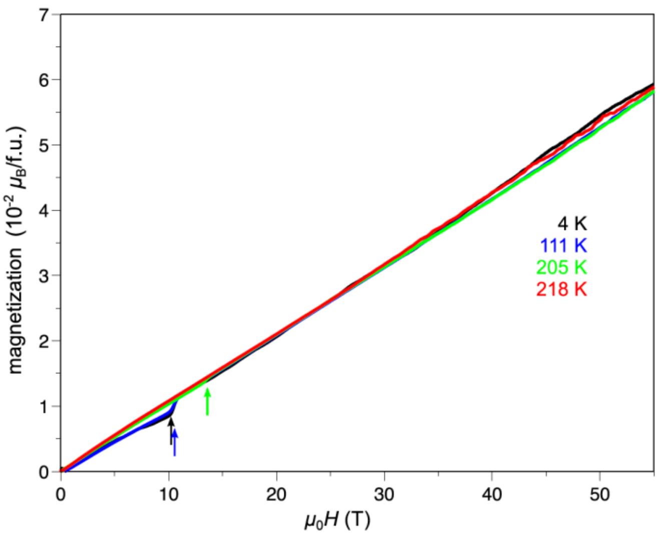


**Supplementary Figure 5│ High field magnetization at some selected temperature.** Magnetization in pulsed magnetic fields (duration of ~30 ms) applied along the *b* axis. The arrows indicate the spin-flop transition from the zero-field AF1 to the high field HF1 phase. The data in pulse fields have been normalized to the DC data measured with SQUID and PPMS.

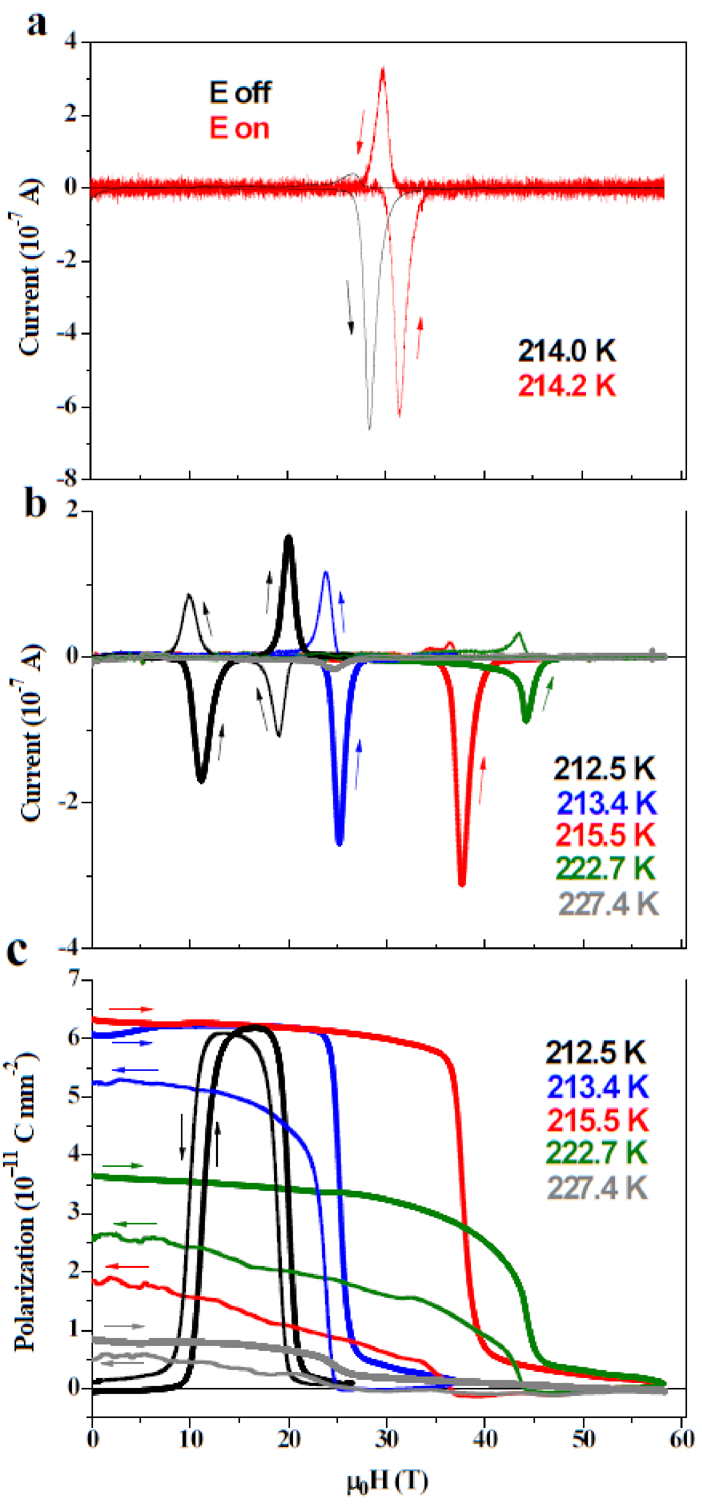


**Supplementary Figure 6 | Extended set of pyrocurrent and polarization data.** (**a**) The pyrocurrent along the *b* axis as a function of **H** applied along the *b* axis measured at about 214 K (within the incommensurate phase) after cooling down from a temperature above 240 K at a polling field of +167 kV/m. In one of the measurements, at 214.2 K, the electrical field was kept "on", while it was turned "off" for the measurement at 214 K. The small difference in the temperature of the two experiments appears due to heating effect of the leakage current during the magnetic pulse; (**b**) the pyrocurrent, and (**c**) the electric polarization along the *b* axis as a function of **H** applied along the *b* axis. Prior each measurement, the pooling field was turned off. The arrows indicate the curves recorded on increasing/decreasing magnetic field during the pulse.

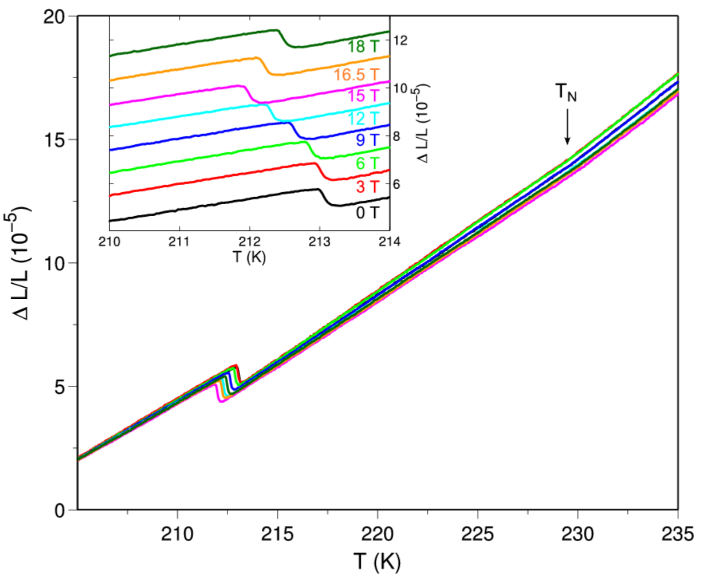


**Supplementary Figure 7│ Thermal expansion.** Temperature dependence of relative length change Δ*L*/*L* along the *b* axis at different DC magnetic fields. The data are recorded on increasing temperature. The data in the inset help to appreciate the magnetic field dependence of the critical temperatures for the AF1→AF2→HF1 border lines determined from the drop in Δ*L*/*L* vs *T* at a certain DC magnetic field. The critical temperature for the AF1→AF2 transition decreases with increasing fields (the curves in the field range 0 to 16 T), while for the HF1→AF2 transition it increases with increasing field (the curves at 16.5 T and 18 T). AF1 represents the low temperature collinear magnetic structure; HF1 - the flopped high field structure at low temperatures; AF2 - the incommensurate oblique helical phase. Please, note that the curves in the inset are shifted vertically by $1\times e^{-5}$ for better visibility.

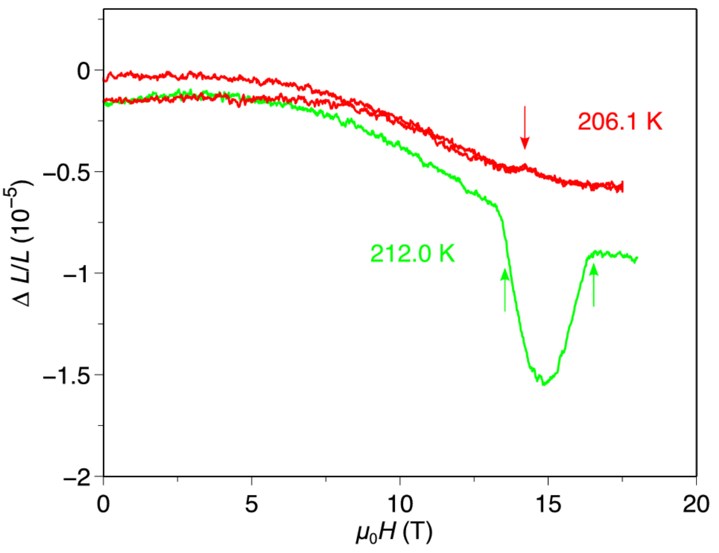


**Supplementary Figure 8│ Magnetostriction.** The relative length change Δ*L*/*L* along the *b* axis *vs* DC magnetic field at two distinct temperatures, 206.1 and 212.0 K. The 206.1 K curve corresponds to AF1→HF1, while the 212.0 K curve illustrates the successive AF1→AF2→HF1 transitions, indicated by arrows. AF1 represents the low temperature collinear magnetic structure; HF1 – the flopped high field structure at low temperatures; AF2 - the incommensurate oblique helical phase.

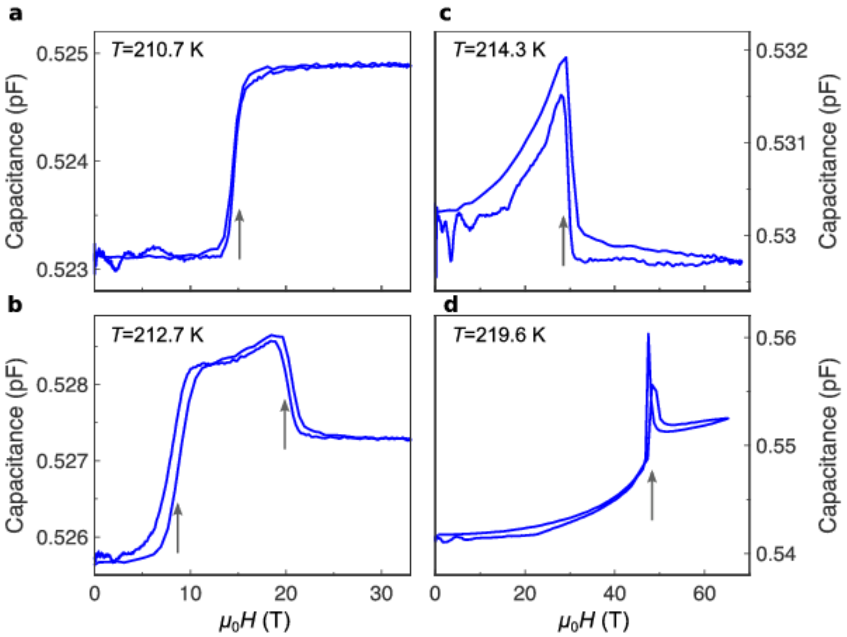


**Supplementary Figure 9│Magnetocapacitance at some selected temperatures**. The arrows indicate the field-induced transitions: AF1→HF1 (at 210.7 K); AF1→AF2→HF1 (at 212.7 K); AF2→HF1 (at 214.3 K); AF2→HF1 or HF3 (at 219.6 K). AF1 represents the low-temperature collinear magnetic structure; HF1 - the flopped high-field structure at low temperatures; AF2 - the incommensurate oblique helical phase; HF3 - the high field, presumably incommensurate sinusoidal collinear structure (the flopped AF3 phase, right below the Néel temperature).

## Supplementary Note 1: Zero magnetic field magnetic structures and transitions:

The sequence of the magnetic transitions at low field is reflected in the temperature dependence of the principle magnetic susceptibility shown in Supplementary Figure 1a and is in agreement with published data[1-3]. Unlike in the conventional 3D paramagnetic state, the susceptibility above $T_{N2} \approx 230$ K increases with increasing temperature in a way reminiscent of low dimensional spin correlations[1], while the anisotropy between different crystallographic directions holds as in the incommensurate phase. As expected[4,5] at the transition from the oblique helical (following the notation of earlier publications we denote for the circular envelope the main axes to be along the crystallographic *b* axis and along an axis within the *ac* plane) to the collinear phase, $T_{N1,}$ the susceptibility along the *b* axis sharply drops, the one along the *c* axis sharply increases, while the *a* axis susceptibility shows a tiny hump. However, unlike in canonical collinear antiferromagnets, the susceptibility along *b* (which is supposed to be the AFM axis below 213 K, i.e., in the collinear phase) does not tend to zero at low temperatures and also the anisotropy between the *a* and *c* axes susceptibilities holds (Supplementary Figure 2). The sequence of the transitions is also seen in the temperature variation of the magnetic torque (Supplementary Figure 1b). The commensurate to incommensurate phase transition is clearly manifested in the temperature dependence of the rocking curves recorded by neutron diffraction at zero magnetic field (Supplementary Figure 1a; see also Supplementary Figure 3). The temperature hysteresis at $T_{N1}$ on cooling/heating runs does not exceed 0.25(5) K on any of the data, in agreement with Ref. 6, while within the experimental resolution no hysteresis was observed at $T_{N2}$.

Both the commensurate and incommensurate zero-field magnetic structures were refined at several selected temperatures, using single crystal neutron diffraction data and applying symmetry analysis to deduce those magnetic moment configurations which are compatible with the nuclear symmetry and the propagation vector. In the commensurate structure [propagation vector *q* = (0.5 0 0.5)], the magnetic moments were confirmed to be collinear and aligned along the monoclinic *b* axis. The ordered magnetic moment at 10 K and 205 K was found to be 0.50(1)$\mu_B$ and 0.37(1)$\mu_B$, respectively. Within the incommensurate phase [propagation vector *q* = (0.506 0 –0.483)], the magnetic moments at 215 K adopt an oblique helix structure with the main axes of the circular (within the experimental errors) envelope along the *b* axis [0.27(1) $\mu_B$] and along an axis within the *ac* plane at an angle $\alpha$ of 35(2)° from the *c* axis towards the positive *a* axis [0.27(1)$\mu_B$]. Note that the sizes of the magnetic moments are in very good agreement with the values given in Ref. 7, but that the angle with the *c* axis presented here is larger than 28.2(8)°. As already pointed out by the authors of Ref. 3, the unpolarized neutron method is relatively insensitive to such an angle especially for non-collinear structures with weak magnetic moments. We therefore would like to stress that our data and refinement qualitatively agree with the superior neutron polarimetry method combined with unpolarized neutron diffraction. The spin-rotation plane makes an angle of 74(2)° with the propagation vector (Fig. 1a of the paper), i.e., the magnetic structure is not a perfect helix (in the sense of moments perpendicular to the propagation vector) as a finite projection of the magnetic moments onto the *q*-*b* plane (the plane containing the propagation vector and the *b* axis) exists resulting in a projected elliptical cycloid (with the main axes 0.27(1) $\mu_B$ || *b* and 0.07(1) $\mu_B$ || $\alpha$. Considering the fact that the spontaneous electrical polarization is found along the *b* axis, it is the projected cycloid, which drives the system to be multiferroic. With increasing the temperature from 215 to 225 K, the total magnetic moment decreases to 0.17(1) $\mu_B$, while the angle of the spin-rotation plane with the *c* axis is constant within the experimental error.

## Supplementary Note 2: Low temperature low field magnetic susceptibility

As mentioned in the Supplementary Note 1, unlike in canonical collinear antiferromagnets, the susceptibility along the *b* axis (which is supposed to be the AFM axis below 213 K) does not tend to zero at low temperatures but remains finite (Supplementary Figure 2).

The above anomalous behaviour might result from several reasons: Our crystals, like the vast majority of the CuO crystals characterized in the literature, might contain a micro-quantity of $Cu^{3+}$ (presumably below 1%), which paramagnetic susceptibility becomes noticeable at low temperatures. Appreciable orbital contribution to the copper moments has been also suggested[4] as a source of the susceptibility anomaly. Some authors[8] argued that a weak ferromagnetism is behind the low temperature susceptibility anomaly, though the neutron diffraction results[7] rule out any appreciable canting.

## Supplementary Note 3: Ultrasound velocity

From the data presented in Fig. 2b of the paper we note that, for all temperatures below about 211.5 K, a double ultrasound velocity anomaly exists at the spin-flop transition between the AF1 and the HF1 phases. We attribute this fine structure to softening of the sound velocity at two magnetic fields of instability, for the low-field AF1 and for the high-field HF1 phases, respectively. In an ideal case of a sample with zero demagnetization factor and absence of intermediate state (a mixture of AF1 and HF1 phases), the two anomalies of the sound velocity should coincide and a single peak at $H_{cr}$ should be observed. However in a real sample the spin-flop transition occurs via an intermediate state, existing within few kOe as our magnetization curves show (Fig. 2a of the paper), that results in a separation of the corresponding fields of instability for the AF1 and HF1 phases and appearance of the double anomaly of the sound velocity. Furthermore, some misalignment of the magnetic field from the *b* axis could provide an additional smoothing of the spin-flop transition that could occur via an angular phase, accompanied by a softening of the sound velocity at the beginning and ending of the spin-reorientation.

The double anomaly manifested in the temperature dependence of the sound velocity, (Supplementary Figure 4) could be explained similarly. The low value of the slop ($dH_{cr}/dT$) evidenced in the inset of Fig. 2a from the paper results in a noticeable temperature separation of the beginning and ending of the spin- reorientation.

## Supplementary Note 4: On the phase coexistence and polar "nucleus"

In general, if the high magnetic field phase is paraelectric and if there are no polar "nucleus" from the low-field phase reminding in it, then no anomaly on the magnetic field dependence of the pyrocurrent, $I(H)$, should be present for the descending branch of the magnetic field $H$ when measured at $E = 0$, i.e., on re-entering the low-$H$ polar phase. We found that this is partially true at temperatures that are within the cycloidal phase but far enough from both, $T_{N1}$ and from $T_{N2}$. For those temperatures, the highest critical fields were measured (see Fig. 3b and the phase diagrams in Fig. 5 of the main text). On approaching those two critical temperatures, the critical magnetic field decreases, the $I(H)$ anomaly for the descending branch of $H$ is enhanced and the difference between the two branches of the electric polarization $P(H)$ diminishes – see, for example, the 212.5 K (for $\mathbf{H} \| b$) or 213.6 K (for $\mathbf{H} \| \alpha$) data in Fig. 3a. We suggest that in the latter cases, the low- and the high-field phases coexist within a broad range of fields above $H_{cr}$, determined by the $I(H)$ minimum/maximum accompanying the sharpest change of $P$ on increasing/decreasing $H$, as also manifested in the magnetic field evolution of the neutron diffraction rocking curves (Fig. 2c of the paper). Actually, it has been revealed by resonant soft x-ray magnetic scattering[9] at zero magnetic field, that multiferroic elongated “nanoregions” (of size $\sim 180 \times 620$ Å$^2$) with finite spin component in the *ac* plane exist within the commensurate AF2 phase of CuO. The authors suggested that the memory effect observed for the direction of **P** on re-entering AF2 trough $T_{N1}$ is due to preserved spin handless since flip of the spin handedness is energetically unfavorable[10]. Broadening of the transition at $T_{N1}$ at zero magnetic field was observed by optical second harmonic generation and attributed to the range of phase coexistence at the first-order transition[11] though the authors called for further experiments to clarify this issue. A similar phenomenon was observed for other magnetically-induced multiferroics, most notably in $MnWO_4$ (Ref. 12). Indeed more experiments are needed to elucidate the nature of the phase coexistence and whether it stems from the character of the first order transition or is a broader demonstration of phase separation phenomena[13], which was found to play an important role in magnetic semiconductors and in the magnetoresistance of the manganites. We speculate that profound magnetoelectric effect could be universal feature of phase-separated regions where polar phase coexists with another phase. Our CuO data show that for such regions even moderate magnetic fields could cause remarkable polarization changes, though in a narrow $T$ interval around the commensurate – incommensurate transition.

## Supplementary references


1. Köbler, U. & Chattopadhyay, T., On the magnetic anisotropy of CuO. *Z. Phys. B - Cond. Matter* **82**, 383-386 (1991).
2. Kimura, T. *et al*., Cupric oxide as an induced-multiferroic with high-Tc. *Nat. Mater.* **7**, 291–294 (2008).
3. Zheng, X.-G. *et al*., Evidence of charge stripes, charge-spin-orbital coupling and phase transition in a simple copper oxide CuO. *J. Phys. Soc. Jpn.* **70**, 1054-1063 (2001).
4. Shimizu, T. *et al*., Spin susceptibility and superexchange interaction in the antiferromagnet CuO. *Phys. Rev*. B **68**, 224433 (2003).
5. Ota, B. &Gmelin, E., Incommensurate antiferromagnetism in copper (II) oxide: Specific-heat study in a magnetic field. *Phys. Rev*. B **48**, 11632 (1992).
6. Babkevich, P. *et al*., Electric field control of chiral magnetic domains in the high-temperature multiferroic CuO. *Phys. Rev.* B **85**, 134428 (2012).
7. Brown, P. J. *et al*., Antiferromagnetism in CuO studied by neutron polarimetry. *J. Phys.: Condens. Matter* **3**, 4281-4287 (1991).
8. Avoni, C. B., Paleari A. & Parravicini, G. B., On the low-temperature magnetic properties of CuO single crystals. *J. Phys: Condens. Matter* **4**, 1359 (1992).
9. Wu, W. B. *et al*., Multiferroic nanoregions and a memory effect in cupric oxide. *Phys. Rev.* B **81**, 172409 (2010).
10. Choi, Y. J. *et al*., Thermally or magnetically induced polarization reversal in the multiferroic $CoCr_2O_4$. *Phys. Rev. Lett.* **102**, 067601 (2009).
11. Hoffman, T., Kimura, K., Kimura, T. & Fiebig, M., Second harmonic generation spectroscopy and domain imaging of the high-temperature multiferroic CuO. *J. Phys. Soc. Jpn*. **81**, 124714 (2012).
12. Urcelay-Olabarria, I. *et al*., X phase of $MnWO_4$. *Phys. Rev.* B **90**, 024408 (2014).
13. Nagaev, E. L., Colossal-magnetoresistance materials: manganites and conventional ferromagnetic semiconductors. *Physics Reports* **346**, 387 (2001).
